# New modalities for managing drought risk in rainfed agriculture: Evidence from a discrete choice experiment in Odisha, India

**DOI:** 10.1016/j.worlddev.2018.03.002

**Published:** 2018-07

**Authors:** Patrick S. Ward, Simrin Makhija

**Affiliations:** International Food Policy Research Institute, Washington, DC, USA

**Keywords:** Drought risk, Insurance, Drought tolerant varieties, India

## Abstract

Droughts have historically had large impacts on agricultural production in rainfed agricultural systems. Scientists and policymakers have proposed various strategies for managing risks, with limited success. In this study we consider two such strategies, specifically drought tolerant rice and weather index insurance. While neither drought tolerant cultivars nor weather index insurance products are perfect solutions for adequately managing drought risk in and of themselves, there is scope to exploit the benefits of each and bundle them into a complementary risk management product, specifically through proper index calibration and an optimized insurance design. In this study, we explore preferences for such a complementary risk management product using discrete choice experiments in Odisha, India. We are able to estimate the added value that farmers perceive in the bundled product above and beyond the value associated with each of the independent products. We also show that valuations are very sensitive to the basis risk implied by the insurance product, with farmers less enthusiastic about risk management products that leave significant risks uninsured.

## Highlights

• This study considers drought tolerant rice and weather index insurance.
• We use discrete choice experiments in Odisha, India.
• We estimate the added value that farmers perceive in the bundled product above.
• We show that valuations are very sensitive to the basis risk implied by the insurance product.
• Farmers less enthusiastic about risk management products that leave significant risks uninsured.

## 1 Introduction

In rainfed rice production systems throughout India, agricultural activities are intimately tied to the arrival and departure of the summer monsoon, and any aberration in monsoon rainfall can have disastrous consequences on foodgrain production, with subsequent impacts on food security and rural livelihoods. Evidence seems to suggest that Indian agriculture may be in the midst of a transition to a new monsoon normal: in five of the last six years, monsoon rains have been weak and unevenly distributed over both time and space, and three of the seven years from 2009 through 2015 have been officially designated as all-India drought years. Total rice production has suffered as a result of these vagaries in monsoon rainfall, both as a result of decreases in harvested area as well as through reductions in rice yields. Many farmers prepare nurseries only after the arrival of the first rains, so any delay in the monsoon onset can result in reductions in total area under cultivation and potentially lower yields. Early monsoon cessation, similarly, can have significant impacts on total rice production, reducing yields due to limited hydration during a critical period in the plants’ development. In addition to these effects arising due to monsoon timing, intermittent, prolonged dry spells during the season can also significantly affect productivity. The drought experienced in 2014 was not as severe as droughts in, for example, 2002 or 2009, yet estimated losses in India were around $30 billion, with national GDP consequently decreasing by about 1.7 percent.

These production effects of drought risk are certainly the most visible effects that droughts impose on Indian agriculture. But in addition to these obvious effects, it is possible that the most substantial effect of droughts in constraining agricultural production in rainfed ecosystems may be through its effect in reducing farmers’ investments in higher risk – higher return activities, such as higher yielding seed varieties and hybrids, using chemical fertilizers, using other crop protection chemicals (e.g., pesticides, herbicides, etc.) and investing in irrigation. In this regard, drought risk can be seen as imposing both ex post as well as ex ante impacts: the ex post impacts are the more visible effects that are only realized after a drought has been experienced; the ex ante impacts are those that are not visible, arising due to merely the threat of drought, but which nonetheless are very real and have sustained impacts. Elbers, Gunning, and Kinsey (2007), for example, suggest that, in sum, combined ex ante and ex post drought risk effects reduce long-term capital accumulation by as much as 46 percent, but nearly two-thirds of this reduction can be attributable to these sorts of ex ante effects. Lybbert and Carter (2014), in turn, refer to droughts as being like “a bully,” where the specter of drought looms over farmers’ heads and compels them to make decisions or take actions that might otherwise be neither rational nor optimal.

The state of Odisha, in eastern India, is often regarded as India’s ‘disaster capital’ owing to the frequency with which it has been victim to droughts, floods, and cyclones over the years. In the 108-year period from 1900 to 2007, Odisha suffered from drought in 39 of those years (Annual Report 2006–7, Government of Odisha). Farmers’ dependency on erratic rainfall in the absence of adequate irrigation facilities has made them extremely vulnerable to the negative ex post impacts of droughts. Pandey, Bhandari, and Hardy (2007) report some of the negative consequences of droughts in Odisha: a 33 percent reduction in gross cropped area and cropping intensity, including a 41 percent reduction in rice area; a 60 percent reduction in rice production; and total income losses of 26 percent. Small and marginal farmers, who constitute the bulk of farmers in Odisha, are often hit the hardest. In addition to these ex post losses from drought, there are significant ex ante losses resulting from drought risk. UNDP (2003) estimates indicate that Odisha faces deficient rainfall (75 percent of normal or less), on average, once in five years. “Knowing that droughts are regular phenomena that cannot be predicted accurately, farmers would have evolved conservative practices that give them some safety even though such practices may result in income losses during normal years” (Pandey et al., 2007, p. 39). Identifying effective means with which to protect farmers from both ex post and ex ante effects of drought risk remains an important objective, both in terms of government policy as well as broader economic and human development.

There are many instruments that can be leveraged to address drought risk. One such intervention involves the breeding of cultivars for drought tolerance and short duration. There has been a great deal of interest within the international donor and agricultural research communities in support of these efforts. In particular, the Stress-Tolerant Rice for Africa and South Asia (STRASA) project, with support from the Bill and Melinda Gates Foundation, has made significant progress in the development of rice varieties tolerant to several different types of abiotic stresses, including droughts, floods, and salinity. The International Rice Research Institute (IRRI), in collaboration with national research partners, has led efforts for developing drought-tolerant (DT) rice varieties suitable for cultivation in drought-prone areas of India, and in 2010 released a DT rice variety (Sahbhagi dhan) for cultivation in Jharkhand, West Bengal, Bihar, and Odisha in eastern India. In experimental trials, this variety has been shown to give better yields over comparison varieties under certain drought stress conditions (Verulkar et al., 2010). Since the variety is still quite new, however, there is still much to be learned about how this variety performs in farmers’ fields.

In what follows, we will assume the following stylized, qualitative characterization of DT yield benefits (c.f. Lybbert & Carter, 2014). Under normal (or irrigated) conditions, there is essentially no yield advantage relative to other commonly grown varieties and indeed may, relative to some high yielding long duration varieties, suffer from a modest yield penalty. Under moderate drought stress, however, the DT variety begins to exhibit yield benefits relative to the non-DT varieties. While absolute yields are still lower than they would be under normal or irrigated conditions, the reduction in yields is considerably less than those observed among the non-DT varieties under the same conditions. In this drought stress region, there are positive and increasing relative benefits associated with DT. Once the drought becomes severe, however, the relative benefits of DT – though still positive – begin to decline.^1^ In other words, while DT yields are still higher than non-DT, the magnitude of the yield advantage is diminishing. Once the drought becomes extreme, the relative benefits of DT are exhausted, and DT is virtually indistinguishable from non-DT. In this region, it can be assumed that both DT and non-DT crops would fail, or would yield so little as to not even justify harvesting. Sahbhagi dhan is also a short duration variety, flowering as quickly as 70–80 days after transplanting and reaching full maturity in less than 100 days after transplanting. This short duration provides a means of escaping either early or late season droughts, for example, arising from delayed monsoon onset or early monsoon cessation.

An alternative intervention that could be used to address drought risk is through providing farmers with insurance. Unlike DT, which primarily addresses ex post drought impacts, insurance is a risk transferal mechanism that helps remove some of the ex ante drought risk burdens. Agricultural insurance, specifically insurance against crop loss, has been around for many years, even in developing countries like India. Pilot crop insurance programs implemented since 1972–73 led to the first major government crop insurance program in 1985–86, the Comprehensive Crop Insurance Scheme (CCIS) that was subsequently replaced by the improved National Agricultural Insurance Scheme (NAIS) in 1999–2000 (Nair, 2010a, Sinha, 2004). These programs used an “area approach”, whereby insurance payouts are made to all farmers in an area where average yields fall below the guaranteed yield (Nair, 2010a). Despite having several national-level programs to promote insurance, however, only about 20 percent of gross cropped area was covered under various insurance schemes as recently as 2014. 2016 saw the launch of the Pradhan Manti Fasal Bima Yojana (PMFBY) which replaced the NAIS.

There are several problems with most of the insurance programs that have been implemented thus far, even the index-based insurance programs. First and foremost is that insurance is typically significantly more expensive than farmers are willing to pay. The cost of insurance factors in not only the risk and magnitude of potential losses, but also administrative loads to compensate for the cost incurred by the insurers in assessing losses as well as risk loads, which are typically higher for agricultural insurance due to the highly covariate nature of the risks being insured against. But the low demand for insurance is not unique to developing countries: even in many developed countries, farmers are typically unwilling to purchase unsubsidized insurance. The subsidies, however, typically impose significant fiscal burdens.

Another problem with traditional crop insurance is that it is susceptible to informational asymmetries, resulting in adverse selection and moral hazard. Weather index insurance (WII) has been promoted as an alternative to traditional indemnity-based agricultural insurance (e.g., Alderman & Haque, 2007), and has gained popularity among development practitioners and researchers in recent years. In the case of WII, the payouts are based on realizations of a specific weather parameter measured over a pre-specified period of time at a particular weather station (World Bank, 2011). Since payouts are ascertained by exogenous information independent of unobservable household characteristics or ex post household decisions (Giné et al., 2008, Karlan and Morduch, 2009, Morduch, 2006), WII partly addresses the problems of moral hazard and adverse selection, as well as some of the delays and high costs of verification present in traditional crop insurance products (Barnett and Mahul, 2007, Carter et al., 2014). But one of the major shortcomings of WII is that, because insurance payments are not directly tied to on-farm performance, there is a possibility that insured farmers could experience a loss and yet not be compensated in the form of an insurance payout. This risk – referred to in the index insurance literature as basis risk – arises because of the spatial-heterogeneity in many of the weather variables on which indices are constructed and the fact that such weather variables may not be particularly strong proxies for on-farm yield and income losses that insurance is meant to protect against (Binswanger-Mkhize, 2012, Michler et al., 2015).^2^ In such cases, there may be years in which the insured has purchased insurance coverage (likely at some premium to the actuarially-fair cost of coverage), experienced a loss, and yet received no compensation, thereby leaving the farmer worse off than if insurance had never been purchased in the first place (Clarke, 2011).

ICICI Lombard, with assistance from the World Bank, first introduced weather-based insurance in Andhra Pradesh, India in 2003 and then in Rajasthan (in collaboration with the state government) in 2004 (Nair, 2010b). In 2004–05, similar weather insurance policies were sold by IFFCO-Tokio and pilot schemes were launched in the states of Andhra Pradesh, Karnataka, and Gujarat (Nair, 2010b). These private sector pilot schemes heralded the entry of public sector insurer, Agricultural Insurance Corporation (AIC) into the weather-based insurance market with the launch of Varsha Bima (rainfall insurance) which had limited coverage until 2007 when the Government of India allocated Rs. 100 crore (Rs. 1 billion) to the Weather-Based Crop Insurance Scheme (WBCIS, Nair, 2010b).^3^ The WBCIS, like the NAIS uses an “area approach”, whereby insurance payouts are made to all farmers in a given area if adverse weather conditions are measured at the weather station in that area (Nair, 2010b). The WBCIS was further restructured in 2016.

There remain, therefore, challenges in providing rainfed farmers with the tools needed to manage production risks, especially – though not limited to – risks associated with droughts. In this paper, we provide evidence on the demand for an innovative risk management tool that takes advantage of the respective strengths of a DT rice variety and WII while simultaneously overcoming some of their individual weaknesses. This bundled DT-WII product results in near comprehensive risk coverage that leverages the inherent complementarities between these two products.^4^

Our primary empirical evidence in this regard comes from a discrete choice experiment used to study preferences over various components of the comprehensive bundle. Our results suggest that, on average, there appears to be significant interest in the DT-WII bundle, with farmers on average willing to pay considerably more for the bundled product than what would be deemed actuarially fair. We also demonstrate heterogeneity in preferences over the various product components. Interestingly, farmers who had been exposed to the DT variety as part of a randomized trial during kharif 2015 had a lower valuation for the DT variety than those who were a part of the comparison group and who had not previously been exposed to the variety. But, while this may signal that such farmers perceive inherent weaknesses in the DT variety, whether due to its slight yield penalty under normal conditions or due to its failure to provide positive yield benefits under extreme drought stress, we note that farmers exposed to the complementary DT-WII product had a significantly higher valuation for the DT variety than those that were only exposed to the DT (i.e., not bundled with the WII), suggesting that the inclusion of the WII in the bundled product increases the perceived value of the DT variety. Across the board, farmers are generally quite sensitive to basis risk, but this sensitivity appears to increase with experience with index insurance. These results suggest scope for promoting a more comprehensive risk management bundle such as the one introduced here, though with the caveat that initial enthusiasm may be tempered with experience, perhaps especially experiences in which insured farmers experience losses but do not receive compensation.

The remainder of the paper proceeds in the following fashion. In Section 2, we introduce the risk management product that results from complementing the benefit profile of a drought tolerant rice variety with a specially-calibrated weather index insurance product, thereby providing absolute benefits (vis-à-vis the non-tolerant variety) under the full spectrum of drought stress. In Section 3, we introduce the data sources and overall data collection procedure, including various experiential learning modules meant to increase farmers’ understanding of complex topics like drought risk, basis risk, and insurance. In Section 3, we introduce the empirical methods used in studying demand for such a risk management bundle, focusing on the discrete choice experiment methodology and estimation procedures allowing for heterogeneous preferences within the population. In Section 4, we report the results of our discrete choice experiment and demonstrate how prior exposure to drought risk management mechanisms can influence preferences and willingness to pay for these mechanisms. Finally, in Section 5, we offer some concluding remarks and discuss policy implications of these research findings.

## 2 Specification of drought risk management product

The individual shortcomings of these two risk management tools offer researchers, practitioners, and policymakers with unique opportunities to learn about the potential interactions between these two tools. Including the two products in a bundled risk management product allows for the product to take advantage of the strengths of each of these tools, allowing for some interesting complementarities (Lybbert and Carter, 2014, Ward et al., 2015). A bundled risk management product consisting of DT rice and a WII product, for example, could provide monotonically increasing (or at least non-decreasing) benefits, since the insurance component would still provide benefits beyond the drought stress level at which the relative benefits of the DT variety begin to decline. Additionally, because the DT variety maintains higher yields than many other varieties under moderate to severe drought stress, the resulting loss in farm incomes during a drought is less than it otherwise would be, implying a smaller amount of farm income at risk due to drought. This has obvious implications for the price of insurance coverage: if less farm income is at risk, or if losses have a lower probability of occurrence, then insurance against these farm income losses should be less expensive.

In this section, we describe the construction and calibration of a bundled DT-WII product for three districts (Balasore, Bhadrak, and Mayurbhanj) in the state of Odisha, in eastern India. Given that the breeding and development of improved varieties (including DT varieties) is often the result of years of investments in research and development by both the private and public sector breeders, it is a much simpler proposal to design an insurance product that is optimized to complement the performance profile of the DT variety than vice versa. As a starting point, we refer to Lybbert and Carter (2014), who helpfully demonstrate how a DT-WII product might be structured. In their paper, they suggest that the insurance product be specified to begin providing benefits at the drought stress level at which the relative benefits of the DT variety start to decline. This approach has appeal, since the relative benefit profile of the complementary product would be monotonically non-decreasing. Indeed, this is the approach that Ward et al. (2015) used in specifying their DT-WII product in their study in Bangladesh. But this approach ignores the reality that, even though the relative benefits may be increasing in moderate droughts, the variety still suffers absolute yield losses under these conditions. In the present study, therefore, we specify our product such that the WII product begins to provide benefits under moderate droughts, with an increased payout under severe drought stress. Since Sahbhagi dhan is a relatively new variety, there is insufficient data on the variety’s performance under a broad spectrum of environmental (including weather) and management conditions. Consequently, insurance optimization is not as straightforward in practice as one might like it to be in theory.

To determine the insurance payments that would be made under moderate and severe drought stress, we first attempt to estimate the value of lost output that farmers in these three districts might expect under these different drought stress levels. Using historical data on district-level rice yields, we estimated a simple linear regression of yields against a time trend that was then used to predict the expected yields under more or less optimal conditions. We then relied upon published estimates (Verulkar et al., 2010) to assume that yield losses under moderate and severe drought stress conditions would be 38 and 69 percent for Sahbhagi dhan. Next, we used historical data on the minimum support price (MSP) for rice in India and extrapolated forward to generate an estimate for the price per kilogram of rice production.^5^ Since the farm gate price is often considerably less than the MSP (e.g., due to transaction or transportation costs), we estimated the value of a kilogram of rice at the farm gate to be 20 percent less than the MSP. With this price in mind, we could estimate the value of lost output under moderate and severe drought stress conditions.

Pricing index insurance requires consideration of the probability that index strike points will be realized and the corresponding payments that will be made if such thresholds are reached, as well as any additional administrative loadings required by the insurer. The two strike points for our rainfall-based WII product were moderate and severe droughts, but as yet we have not explicitly described how we defined “drought.” Droughts have to do with rainfall deficiencies, but are complex phenomena arising due to meteorological, hydrological, and other factors. While a drought could simply be a deviation in cumulative rainfall over the course of the entire season relative to long-term averages, the most obvious form of drought is perhaps the occurrence of a prolonged dry spell during the course of the season. Because prolonged dry spells can be classified as extreme weather events, it is appropriate to appeal to Extreme Value Theory (EVT) and model these extrema using an extreme value distribution.^6^ There are two primary approaches to analyzing extremes. The first approach is to analyze excesses over a sufficiently high threshold, an approach often referred to as “peaks-over-thresholds” or “threshold exceedance.” The second approach analyzes the extrema of long blocks of data, for example, annual maxima. This latter approach reduces the data considerably, since you are left with only one observation per block (e.g., year). Since our primary purpose in the present study is to understand farmers’ willingness-to-pay for a bundled DT-WII product, and since we only have information on the longest dry spell that the DT variety could withstand, we preferred to structure our complementary insurance component based upon the latter approach, which allows us to assess the probability that an extremely long dry spell would be observed in any given year. The generalized extreme value (GEV) distribution function takes the form

(1) $$F(x\text{;}\xi \text{,}\alpha \text{,}\kappa )=\exp\left\{-{\left[1+\kappa \left(\frac{x-\xi }{\alpha }\right)\right]}^{-1/\kappa }\right\}\text{,}$$

where x is some datum (in our specific case, the annual maximum monsoon season dry spell for a particular year), ξ∈R is the location parameter, α>0 is the scale parameter, and κ∈R is the shape parameter. Clearly, F(x;μ,σ,ξ) yields the probability that the longest monsoon season dry spell is less than or equal to x, and thus $p(x^{*}⩾x)=1-F(x\text{;}\mu \text{,}\sigma \text{,}\xi )$. These parameters can be estimated using maximum likelihood, and the estimates can be used to determine return levels, return periods, and the probability of an extreme event occurring.^7^ If the set $\left\{x_{i}\right\}$ is the sequence of annual maximum monsoon season dry spells, which is assumed to be independent and identically distributed from a GEV distribution, then the log-likelihood function for a sample of *n* observations $\left\{x_{1}\text{,}x_{2}\text{,}\ldots \text{,}x_{n}\right\}$ is^8^

(2) $$\text{ln}[L(\xi \text{,}\alpha \text{,}\kappa |x)=\overset{n}{\underset{i=1}{\sum }}\left\{-\ln\alpha -\left(1+\frac{1}{\kappa }\right)\ln\left[1+\kappa \left(\frac{x_{i}-\xi }{\alpha }\right)\right]-{\left[1+\kappa \left(\frac{x_{i}-\xi }{\alpha }\right)\right]}^{-\frac{1}{\kappa }}\right\}\text{.}$$

By maximizing this log-likelihood function, we obtain estimates for the district-specific location, scale, and shape parameters characterizing the distribution of these maxima. With estimates ξ^,α^, and κ^, we can then estimate the event $x_{p}$ that will occur with probability p. To solve for this, we simply write:

(3) x(p)=μ^-σ^κ^{1-[-ln(1-p)-κ^]}.

For example, if we wanted to know the severity of a 100-year event (i.e., a maximum dry spell that would be expected to occur only once every 100 years), we would simply use p=0.01 and solve for x. Based on figures reported in Kar et al. (2004), the probability of $I_{i}$ a ‘moderate drought’ in Balasore and Mayurbhanj is 26.67 percent and 20.94 percent, respectively, while the probability of a ‘severe drought’ in Balasore and Mayurbhanj is 2.23 percent and 2.33 percent, respectively.^9^ In other words, ‘moderate droughts’ occur in these districts roughly once every four or five years, while ‘severe droughts’ occur roughly once every 40 years.

Actuarially-fair insurance is priced such that the cost of insurance equals the expected payout received. Consider a simple index insurance product with discrete strike points, i=1,…,n, and let $p_{i}$ define the probability of strike point i being triggered. Let be monetary amount of the insurance payout under strike point i. Then an actuarially fair premium would be $A=\sum_{i=1}^{n}p_{i}I_{i}$. Of course, since the actuarially fair cost of insurance reflects an insurers expected payouts, in the long run insurers should not expect to make any money, resulting in an unsustainable business model. Furthermore, since weather risk is primarily a covariate risk, insurers generally must be compensated for the risks they are exposed to in insuring many people who all share the same covariate risk. To address both of these concerns, we assume a 30 percent premium on to the actuarially fair cost of the insurance to cover both risk and administrative loads. In reality, this is considerably lower than what insurance companies would likely add, but since this product was designed for a pilot program in a part of the world with very little experience in insurance, we were reluctant to charge more than just this modest premium. Details on the cost and structure of these insurance products across the three districts are shown in Table 1. This merely reflects the insurance component of the DT-WII bundle. The actual cost of the bundled product would also need to reflect the INR 80 for the 2 kg bag of seed.

**Table 1:** Specification of rainfall-based weather index insurance product to complement drought tolerant rice variety, by district.

			Moderate drought		Severe drought	
	Actuarially-fair cost	Cost inclusive of 30 percent premium	Dry spell length	Payout	Dry spell length	Payout
Balasore	100	130	18	330	27	600
Bhadrak	115	150	18	370	31	670
Mayurbhanj	75	100	20	300	25	540

Recall our earlier assumption (drawn from published estimates in Verulkar et al., 2010) that Sahbhagi dhan yields decline by 38 and 69 percent under moderate and severe drought stress, respectively. These yield reductions are considerably less than the yield reductions for Swarna, the most commonly grown variety in our sample area (58 and 89 percent under moderate and severe drought stress, respectively). Assuming that the two varieties produce a homogeneous output that can be sold at the same market price, this implies that cultivating Sahbhagi dhan results in smaller overall crop production losses that would need to be insured. This illustrates how the DT component provides an implicit subsidy on the cost of insurance. In this case, the subsidy is roughly INR 50 in Balasore, INR 56 in Bhadrak, and INR 35 in Mayurbhanj, representing a 32–33 percent reduction on the actuarially fair cost of insuring farm income losses from drought. This does not even consider the fact that bundling the drought tolerant variety with WII also reduces the insurer’s value-at-risk, which has significant implications for the cost of reinsurance, much of which might also be passed on to the insured in the form of higher administrative loads on top of the actuarially fair cost. Given that many insurance pilots have met such limited success due to insufficient demand, and given many governments’ and development practitioners’ preferences against providing explicit subsidies (not to mention most neoclassical economists’ disdain for subsidies), this implicit subsidy may be a powerful tool for providing the incentives necessary to promote widespread adoption of the drought tolerant-WII risk management product.^10^

## 3 Data

The data reported on in this paper are drawn from a survey conducted in the three districts of Balasore, Bhadrak, and Mayurbhanj in Odisha during February–March 2016. The survey served as the midline survey for a multi-year randomized evaluation on the impacts of a specially-calibrated drought risk management bundle like one introduced in Section 2. As part of the larger evaluation, our pool of respondents consists of a total sample of 2160 respondents drawn from a total of 111 villages across three districts in the state of Odisha, India. Since part of the evaluation would involve actually marketing the risk management product, one of the first steps in establishing our sample frame was to identify the appropriate partner. To this end, we established a relationship with Balasore Social Services Society (BSSS), a nongovernmental organization operating in several districts in Odisha. BSSS proved to be an ideal partner in many ways, since they have experience in assisting with other research projects (including other randomized evaluations), they had already-established relationships with other initiatives managed by international agricultural research organizations (including the STRASA project described earlier, which facilitated relatively easy access to seed supplies), and the pilot project objectives were well within their organizational mandates.

**Fig. 1:**
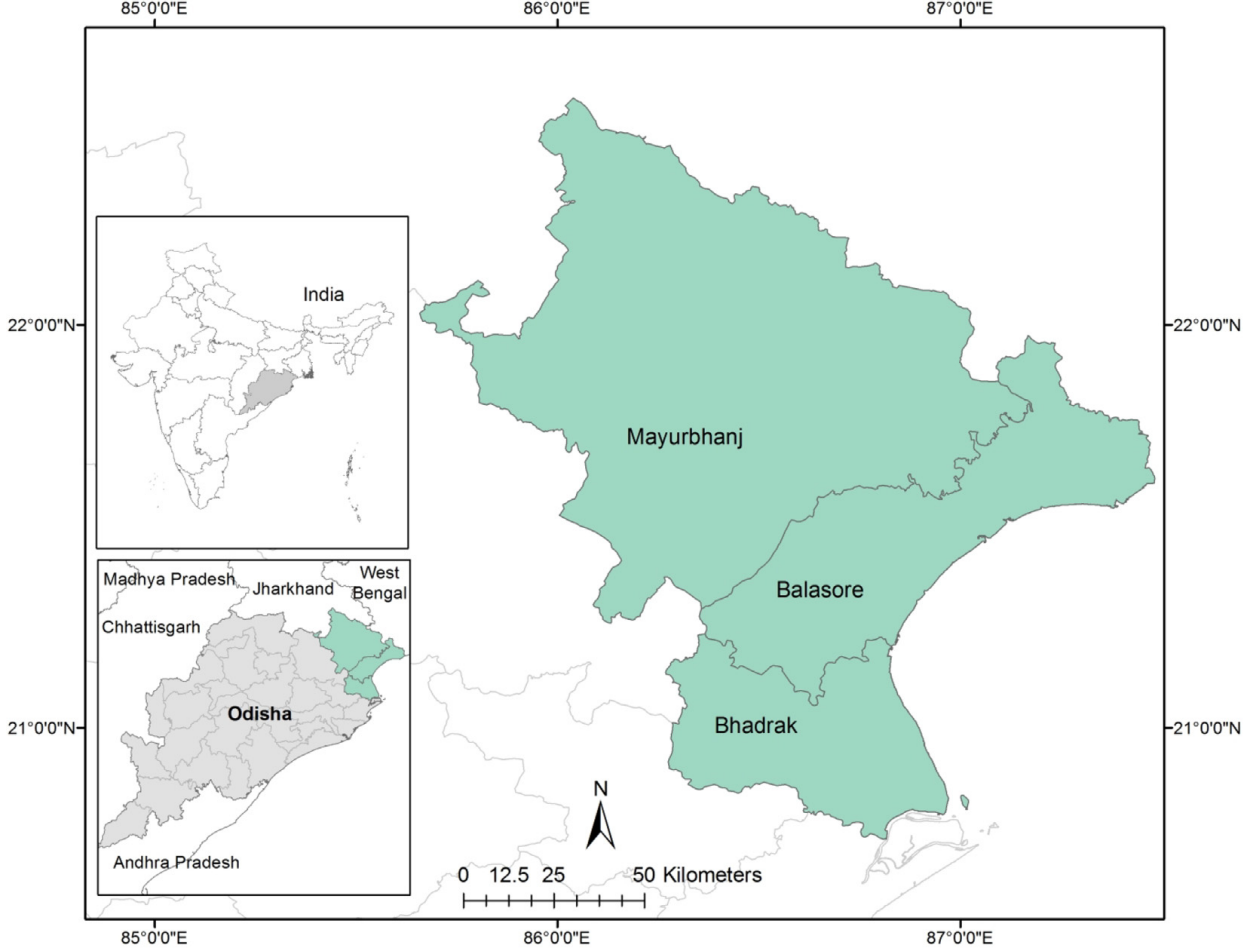
Map of districts in study area.

Since BSSS worked in three districts (Balasore, Bhadrak, and Mayurbhanj), these became our focal districts. The population of interest comprises the region at the intersection of these three districts (see Fig. 1 below). This region includes the six blocks (sub-district administrative units) of Khaira and Oupada in Balasore, Bant and Agarpada^11^ in Bhadrak and Kaptipada and Thakurmunda in Mayurbhanj. This region was identified for the study as farmers in this region predominantly grow rice under rainfed conditions and are considered extremely prone to drought.

From these six blocks, we selected 111 sample villages using probability proportional to size (PPS) sampling, with populations drawn from the 2011 Census. These 111 villages were then randomly assigned to three different experimental arms (2 treatment and 1 control arm). In one of the treatment arms, villages were only offered Sahbhagi dhan, while in the other treatment arm, villages were offered the DT-WII bundle described above. While the 2011 Census data served as the basis for village selection, we were also cognizant that village populations sometimes undergo significant changes, particularly in parts of India like Odisha that have frequent seasonal and interseasonal migration. In order to avoid even the risk of systematically omitting households who may have moved into these villages in the intervening years following the Census, we used a multi-step approach to collating village lists from which participant households would ultimately be drawn. First, pairs of enumerators visited each village to obtain the most recent list of households residing in that village. They were instructed to collect these lists by either writing down a list of all households as verbally listed by the village head or another figure of authority in the village; collecting a copy of the 2011 census (2011) or Below Poverty Line (an official designation) listing and asking the village head or another figure of authority in the village about whether any of the households listed were no longer residing in the village or deceased; or additionally, writing down names of households that have moved to the given village since the census. Given the nature of our pilot, we employed two selection criteria to identify suitable households: households had to own land that was used for agricultural purposes (i.e., they could not be purely tenant households) and households had to cultivate rice. For each of the 111 villages, a random sample of 30 households was drawn from this pool of eligible households. From this list of 30, 20 households were then selected at random to serve as the primary sample, with the remaining 10 serving as replacement households that would be drawn upon if any of the households in the primary sample were either missing at the time of interview or otherwise subsequently determined ineligible to participate in the study.

The midline household survey that serves as the basis for this paper contained many of the usual survey modules that appear in agricultural development research, including detailed agricultural production modules, food and non-food consumption modules, household assets, etc. The survey also contained specialized modules that allow us to evaluate interventions that were introduced during the first year of the study (kharif 2015). These include specific modules targeted toward respondents in the two treatment arms that cover experiences with the risk management products they were offered. These experiences provide an additional layer of farmer heterogeneity that will be explored when evaluating differences in willingness-to-pay for and actual uptake of these risk management products.

## 4 Empirical methods

To study farmers’ preferences for drought risk management products, our study relies upon choice modeling techniques. Specifically, our study uses discrete choice experiments, a form of stated choice analysis in which preferences are elicited from participants based upon responses to hypothetical scenarios rather than observed choices in actual market settings. The advantage of such an approach is that it allows the researcher to study stated preferences in settings where, for example, functional markets are sparse or nonexistent, or when it may be beneficial to consider the implications of multidimensional policy changes. DCEs provide a theoretically consistent methodology for evaluating stated preferences within an experimental environment controlled by the researcher. In DCEs, individuals are presented a series of choice scenarios comprised of hypothetical products, each described by a set of product traits (or attributes). Researchers control the experimental choice environment by providing necessary variation in attribute levels, which may not be present in historical data (in other words, the random variation in attribute levels permissible in a DCE might not exist for analysis of preferences revealed through real-world purchases). By employing statistical techniques to analyze data characterizing participants’ choices in a particular choice task given the characteristics of the choice alternatives available, the analyst is able to estimate marginal preferences for the various attributes embodied in the alternatives.

In the present study, the agents under analysis are farm producer-consumers. We assume that household production and consumption decisions are non-separable – and thus made simultaneously – which allows us to incorporate production-side traits within the framework of random utility theory, which describes discrete choices as arising from utility maximization (McFadden, 1974). Suppose that individual i is faced with a production-related decision consisting of J alternatives during a particular choice scenario t. We can define an underlying latent variable $v_{\mathrm{ijt}}^{*}$ that denotes the utility associated with individual i choosing alternative j∈J during t. This utility is comprised of a deterministic component, in which individual tastes and preferences map the attribute expressions directly into utility, and a random component, reflecting unobserved, idiosyncratic variations in taste, errors in optimization, etc. Combining the deterministic and random components, we can express utility as

(4) $$v_{\mathrm{ijt}}^{*}=x_{\mathrm{ijt}}^{\prime }\beta_{i}+\varepsilon_{\mathrm{ijt}}$$

where, in this case, $x_{\mathrm{ijt}}$ is the vector of attributes observed by individual i within alternative j during choice scenario t, $\beta_{i}$ is a vector of individual-specific preferences or taste parameters (i.e., a vector of weights mapping attribute levels into utility), and $\varepsilon_{\mathrm{ijt}}$ is a stochastic component of utility, which is independent and identically distributed across individuals and alternative choices. While each individual in the population has unique preferences, we assume that individual preferences are randomly distributed in the population, such that $\beta_{i}∼f(\beta |\Omega )$, where the vector Ω defines the parameters characterizing the distributions of preferences within the population (e.g., mean and variance in the case of the normal distribution).

With observed choices $y_{i}=(y_{i1}\text{,}\ldots \text{,}y_{\mathrm{iT}})$, we can estimate the probability that a particular vector of preferences would yield the observed choice. Since the error terms are Gumbel distributed, the difference between the two error terms takes a logistic distribution. If individual preferences $\beta_{i}$ were known, then probability of the observed sequence of choices–conditional upon the attribute levels observed in the choice scenarios–would simply be the product of T conditional logits. Since we do not know $\beta_{i}$, however, the conditional probability of the observed sequence is estimated by integrating over the distribution of possible preference vectors:

(5) $$P(y_{i}|x_{i1t}^{\prime }\text{,}x_{i2t}^{\prime }\text{,}\ldots \text{,}x_{\mathrm{iJt}}^{\prime }\text{,}\Omega )=\int \frac{\text{exp}(x_{{\mathrm{iy}}_{\mathrm{it}}t}^{\prime }\beta_{i})}{\sum_{q=1}^{Q}\text{exp}(x_{\mathrm{iqt}}^{\prime }\beta_{i})}f(\beta |\Omega )d\beta$$

This is the mixed (or random parameters) logit model (McFadden and Train, 2000, Revelt and Train, 1998, Revelt and Train, 1999, Train, 2003), which is a generalization of the conditional logit model (McFadden, 1974). The mixed logit model is regarded as a highly flexible model that can approximate any random utility model and relaxes the limitations of the traditional conditional logit by allowing random taste variation within the population (McFadden and Train, 2000, Train, 2003). Because the integral in Eq. (5) will not generally have a closed form solution, the conditional probability can be approximated by maximum simulated likelihood estimation, where the simulations are typically based on a large number of Halton draws.

Operationalizing estimation of Eq. (5) requires some specification. Specifically, we must specify the family of distributions from which each of the random parameters are drawn. Typically, researchers allow for most parameters to be distributed normally. This has intuitive appeal, but, since the domain of the normal distribution is infinite, it introduces the possibility of extreme values, which can exert excessive influence on the population distributions and calculations of WTP. In our empirical application, we allow all non-price preferences to be distributed according to a truncated normal distribution: $\beta_{\mathrm{ij}}=\beta_{j}+\sigma_{j}\nu_{\mathrm{ij}}$, where $\nu_{\mathrm{ij}}$ is mean-zero and normally distributed on (−1.96, 1.96).^12^ This maintains the intuitive appeal of normally distributed preferences in the population, while imposing some moderate restrictions on parameter space for the underlying empirical distribution. We allow the (negative) price coefficient to take a one-sided triangular distribution: $\beta_{\mathrm{iP}}=\beta_{P}+\beta_{P}\nu_{\mathrm{iP}}$, where $\nu_{\mathrm{iP}}$ has a triangular distribution with limits (−1,1). This assumption yields the intuitive and theoretically consistent result that the marginal utility of income (marginal disutility of price) is always non-negative (non-positive), while also restricting $\beta_{\mathrm{iP}}\in [-2\beta_{P}\text{,}0)$. Furthermore, the shape of the distribution is such that it crudely approximates normality, with a similar pattern of central tendency.^13^

Given that our sample consists of farmers with different experiences with the risk management products under consideration (i.e., some farmers were exposed to the DT variety in kharif 2015; some were exposed to the DT-WII product and cultivated the DT variety in kharif 2015; some were in the comparison group and were not afforded the option of purchasing either of these products), we will ultimately be interested in studying how preferences for these products vary based on these experiences. To facilitate such comparisons, we will estimate Eq. (5) separately for each group. But given the possibility for differences in scale across sub-samples (Swait & Louviere, 1993), the preference coefficients estimated in each model will not be directly comparable. Rather, we must transform the estimates into a common unit that can facilitate such comparisons. To this end, we will convert preferences into a monetary measure that is purged of the potential influence of the unknown scale parameter. If $\beta_{\mathrm{iP}}$ can be interpreted as the marginal (dis-) utility of product price (which we assume to be negative, consistent with standard economic theory), and assuming that “a penny saved is a penny earned,” the inverse of the marginal disutility of price is simply the marginal utility of money or income. With an estimate for the marginal utility of money, the marginal rate of substitution of money for each of the corresponding attributes – that is, willingness-to-pay (WTP) – can be estimated. Since WTP is the ratio of two random variables, WTP has a probability distribution of its own. We will therefore be interested in both the mean and the variance of individuals’ WTP for the different bundle components. We can approximate the mean and variance WTP by taking first-order Taylor Series approximations around the population means, which are the posterior mean estimates that result from estimating Eq. (5). It can easily be shown that, given the distributional assumptions specified above,

(6) $$E[{\text{WTP}}_{\mathrm{ij}}]≅\frac{E[\beta_{\mathrm{ij}}]}{E[\beta_{\mathrm{iP}}]}$$

(7) $$\text{Var}[{\mathrm{WTP}}_{\mathrm{ij}}]≅\frac{E{\left[\beta_{\mathrm{ij}}\right]}^{2}+\sigma_{\mathrm{ij}}^{2}}{E{\left[\beta_{\mathrm{iP}}\right]}^{2}}\text{.}$$

The marginal WTP for favorable (unfavorable) attributes will be positive (negative). Obviously, if there are interaction terms included in the utility function, this expression will be slightly different, but such modifications are straightforward: the numerator will simply be the partial derivative of indirect utility with respect to the particular attribute.

The implementation of the discrete choice experiment in the field was similar in many ways to that of Ward et al. (2015), including many of the attributes that comprised the hypothetical alternatives. Following previous research (Dalton and Muhammad, 2011, Ward et al., 2014, Ward et al., 2015), we treated the DT varieties’ inherent drought tolerance as a yield distribution, with different yields under normal conditions, moderate drought stress, severe drought stress, and extreme drought stress. The actual yields that were reported are based on experimental trials reported in Verulkar et al. (2010). While there are four yield levels reported for each variety, we treat this attribute as binary, with values of one corresponding to a DT variety (Sahbhagi dhan) and values of zero corresponding to the most commonly grown variety in Odisha (Swarna).

We also included an attribute corresponding to the duration from transplanting to maturity, since Sahbhagi dhan’s short duration provides an additional form of drought protection, allowing farmers to delay transplanting (in the case of a delayed monsoon) or harvest their rice crop early (which would allow them to escape an early monsoon cessation). Furthermore, short duration potentially enables farmers to cultivate a crop after harvesting their rice using the residual soil moisture.^14^ On the other hand, many farms in Odisha consist of relatively small, disconnected plots, so growing a short duration variety on a plot surrounded by long duration rice can complicate harvesting, especially if one wants to use mechanical harvesters. In our experiment, we allowed for the duration attribute to take one of two levels: short (less than 135 days) or long (longer than 135 days).

The choice experiment also included an insurance component. While in practice the insurance component of the potential bundle would be constructed as identified in Section 2, in the choice experiment we used a simplified version that simply reported the insurance payouts under moderate and severe drought stress conditions, without explicitly identifying the conditions under which these payments were triggered. This attribute consisted of two levels, one corresponding to an insurance product intended to complement the performance profile of the drought tolerant rice variety (i.e., a limited coverage insurance policy), and one that was essentially a standalone insurance product meant to compensate for income losses for farmers cultivating non-tolerant varieties (i.e., a full coverage insurance policy). The limited coverage policy would pay out INR 340 under moderate drought conditions and INR 610 under severe drought conditions, while the full coverage policy would pay out INR 510 under moderate drought conditions and INR 780 under severe drought conditions.

We included an additional attribute corresponding to basis risk. When introducing the choice alternatives, this attribute was colloquially couched as the probability of not receiving a payment even when you experience a drought, though admittedly this is an oversimplification of basis risk. Prior to commencing the choice experiment, enumerators read through a series of experiential learning scripts, including one explicitly intended to provide respondents with a cursory introduction to the concepts of basis risk. This attribute took two levels, one corresponding to 30 percent basis risk (i.e., there is a 70 percent chance that one would receive an insurance payment after experiencing a drought) and one corresponding to 0 percent basis risk (in which one would always receive a payment if they experienced a drought).

The final attribute included in the choice experiment corresponds to the bundle price. As demonstrated above, including a monetary attribute in the choice experiment affords us the opportunity to compute a measure of willingness to pay, which is a pecuniary measure of wellbeing that has direct interpretations for wellbeing. We included four monetary attributes, meant to reflect a wide spectrum of potential risk management products, ranging from seed only through a combined purchase of both seed as well as a full coverage weather index insurance product. The prices included in the choice experiment were INR 40, INR 80, INR 160, and INR 200. While these prices were presented to farmers as discrete variables, the price attribute was treated as a continuous product attribute when analyzing the choice data.

**Fig. 2:**
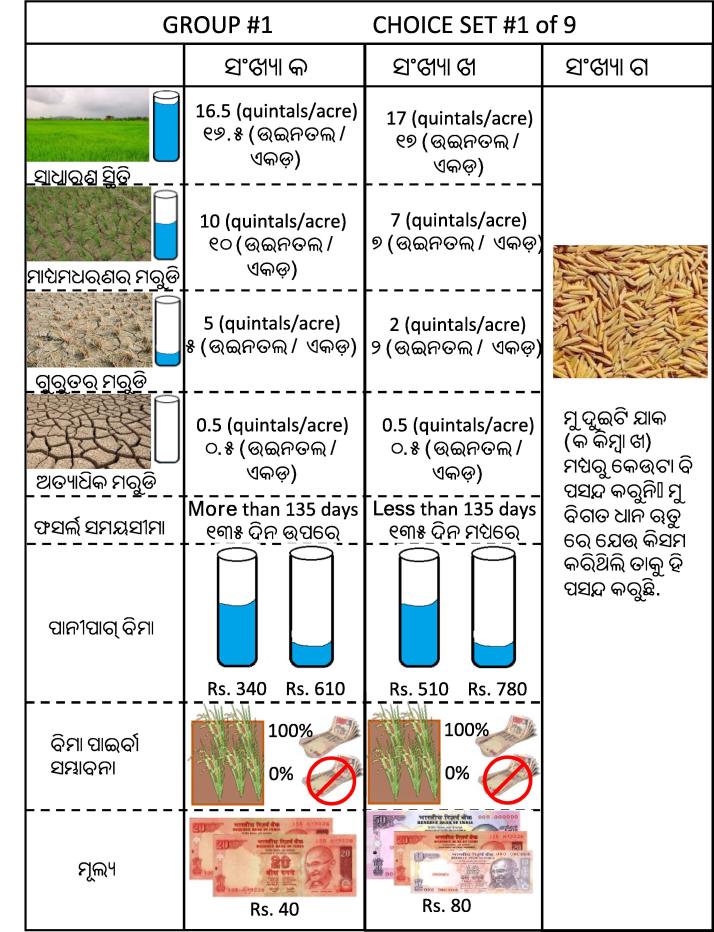
Example of choice card presented to farmers.

Choice sets were constructed using Ngene software v. 1.1.2 (ChoiceMetrics, 2014) based on D-optimality design criteria. The experimental design was based on quasi-informative priors derived from a combination of both previous research (e.g., Ward et al., 2015) and analysis of pre-test data. The ultimate design consisted of 36 unique choice sets, which were randomly assigned to four different choice set blocks, such that each respondent was required to respond to nine choice sets. Each of the study participants were randomly allocated to these four blocks. An example choice set is shown in Fig. 2.

## 5 Results

Estimation results for the choice model using a conditional logit and mixed logit specifications are presented in Table 2 below. These estimate the posterior mean marginal utilities and posterior mean standard deviations that are estimated via simulated maximum likelihood. The marginal utility estimates provide insight into relative preference orderings, while the standard deviations provide insight into preference heterogeneity (i.e., how widely preferences are distributed throughout the population). In column (1), we report the estimates for the full sample, while in columns (2)–(4), we report the estimates for the various experimental arms from the randomized evaluation described above.

**Table 2:** Random Parameters logit and mixed logit estimates of choice model.

	(1)	(2)	(3)	(4)
	Pooled sample	DT treatment arm	DT-WII treatment arm	Control arm
***Marginal utility estimates***				
Drought tolerant Yield	3.550^***^	2.844^***^	2.975^***^	4.261^***^
	(0.131)	(0.198)	(0.200)	(0.288)
Short duration	0.175^***^	0.103	0.195^**^	0.123
	(0.047)	(0.084)	(0.081)	(0.084)
Full coverage	5.167^***^	5.239^***^	4.683^***^	5.173^***^
	(0.103)	(0.176)	(0.157)	(0.18)
Limited coverage	4.462^***^	4.561^***^	4.179^***^	4.373^***^
	(0.090)	(0.153)	(0.141)	(0.152)
Basis risk	−6.058^***^	−5.795^***^	−6.140^***^	−6.030^***^
	(0.141)	(0.242)	(0.237)	(0.235)
Negative cost	1.696^***^	1.890^***^	1.454^***^	1.778^***^
	(0.042)	(0.079)	(0.064)	(0.074)

***Distributions of random parameters***				
SD (Drought tolerant yield)	4.25^***^	4.005^***^	4.001^***^	5.257^***^
	(0.132)	(0.213)	(0.218)	(0.291)
SD (Short duration)	1.632^***^	1.743^***^	1.646^***^	1.632^***^
	(0.070)	(0.118)	(0.115)	(0.12)
SD (Full coverage)	2.099^***^	1.893^***^	1.810^***^	2.128^***^
	(0.075)	(0.125)	(0.123)	(0.142)
SD (Limited coverage)	1.397^***^	0.894^***^	1.283^***^	1.337^***^
	(0.078)	(0.166)	(0.120)	(0.180)
SD (Basis risk)	3.615^***^	3.791^***^	3.702^***^	3.277^***^
	(0.143)	(0.247)	(0.242)	(0.248)
SD (Negative cost)	1.696^***^	1.890^***^	1.454^***^	1.778^***^
	(0.042)	(0.079)	(0.064)	(0.074)

Number of households	2,160	695	720	745
Number of choice sets per household	9	9	9	9
Total number of observations	19,440	6,255	6,480	6,705
Number of parameters (K)	11	11	11	11
Log likelihood	−12,598.88	−4,154.23	−4,287.24	−4182.51
McFadden pseudo-R^2^	0.410	0.395	0.397	0.432

Note: ^***^ significant at 1% probability of type I error; ^**^ significant at 5% probability of type I error; ^*^ significant at 10% probability of type I error. Standard errors in parentheses. Random parameters logit model estimated using NLOGIT 5.0 based on 1000 Halton draws used for simulated maximum likelihood. Models assume non-price main effect marginal utility coefficients are distributed according to truncated normal distribution, while price coefficient is assumed to be distributed according to a one-sided triangular distribution.

Based on the results across the different samples, we see that there is, on average, a statistically significant positive marginal utility associated with the DT yield distribution, though the estimated deviation parameters also suggest a great deal of heterogeneity in preferences (the derived coefficient of variation on preferences in the population is 1.2). We also find that there are positive and statistically significant marginal utilities for each of the two insurance products (the full coverage policy and the limited coverage policy). While there is evidence of preference heterogeneity within each of the experimental arms and in the broader population, the variation in preferences in each of these samples is incredibly small relative to the utility coefficient. Not surprisingly, we also find strong evidence of significant disutility associated with basis risk, a result that holds across both the pooled sample as well as each of the experimental treatment arms. While the marginal utility for the short duration is statistically significant in the pooled sample and the DT-WII treatment arm, the magnitude of the coefficient is so small (especially relative to the other utility coefficients), that short duration appears to be economically insignificant. In other words, it is not the case that farmers prefer long duration, but rather it appears as though farmers do not have a strong inclination one way or the other. Indeed, the derived coefficient of variation for this attribute is greater than 8 in each of the different subsamples, suggesting that the attribute may be largely ignored when considering drought risk management (e.g., Hess and Hensher, 2010, Ortega and Ward, 2016).

Because utility is a non-cardinal measure, these results are not easily interpretable beyond providing information on preference rankings, and due to potential differences in scale that emerge across samples, it is not possible to make any sort of comparisons across samples from this table. It is more informative to examine the monetary valuations of these traits achieved by calculating the mean marginal WTP based on Eq. (7). These are reported in column (1) of Table 3, with 95 percent confidence intervals for these estimates in column (2). In columns (3)–(5), we report t-statistics from comparisons of the estimated WTP for each attribute across the different samples. For example, in column (3), we report the t-statistics for the differences in the estimated WTP for each attribute between the DT treatment arm (Panel 2), the DT-WII treatment arm (Panel 3), and the control group (Panel 4) and the pooled sample.

**Table 3:** Empirical estimates of marginal willingness to pay (INR 100) for risk management attributes from random parameters logit model, across experimental treatments.

			(1)	(2)	(3)	(4)	(5)
			WTP Coefficient	95% Confidence Interval	Δ_Pooled_	Δ_DT_	Δ_DT/WII_
***Panel 1***							
Pooled sample	WTP						
		DT	2.093^***^	[1.934, 2.251]			
		Short duration	0.103^***^	[0.049, 0.158]			
		Full insurance	3.046^***^	[2.933, 3.159]			
		Limited insurance	2.630^***^	[2.536, 2.724]			
		Basis risk	−3.571^***^	[−3.738, −3.404]			

***Panel 2***							
DT treatment arm	WTP						
		DT	1.505^***^	[1.293, 1.718]	−42.218^***^		
		Short duration	0.055	[−0.033, 0.142]	−5.540^***^		
		Full insurance	2.772^***^	[2.608, 2.936]	−22.564^***^		
		Limited insurance	2.414^***^	[2.278, 2.549]	−19.584^***^		
		Basis risk	−3.067^***^	[−3.321, −2.813]	33.545^***^		

***Panel 3***							
DT-WII treatment arm	WTP						
		DT	2.046^***^	[1.760, 2.332]	−2.992^***^	28.555^***^	
		Short duration	0.134^**^	[0.024, 0.244]	3.219^***^	6.654^***^	
		Full insurance	3.222^***^	[3.007, 3.436]	13.132^***^	27.211^***^	
		Limited insurance	2.874^***^	[2.684, 3.065]	19.479^***^	30.063^***^	
		Basis risk	−4.224^***^	[−4.583, −3.865]	−38.091^***^	−55.113^***^	

***Panel 4***							
Control group	WTP						
		DT	2.397^***^	[2.068, 2.725]	18.777^***^	45.666^***^	16.950^***^
		Short duration	0.069	[−0.024, 0.162]	−3.916^***^	1.275	−5.458^***^
		Full insurance	2.910^***^	[2.727, 3.093]	−11.034^***^	8.787^***^	−18.708^***^
		Limited insurance	2.460^***^	[2.313, 2.607]	−15.316^***^	3.286^***^	−26.977^***^
		Basis risk	−3.392^***^	[−3.656, −3.127]	12.040^***^	−16.958^***^	39.854^***^

Note: ^***^ significant at 1% probability of type I error; ^**^ significant at 5% probability of type I error; ^*^ significant at 10% probability of type I error. Confidence intervals based on standard errors computed using the delta method. Differences reported in columns represent differences in estimated WTP relative to pooled sample, DT-only experimental treatment arm, and DT-WII experimental treatment arm, respectively.

In Panel 1 of Table 3 we see that, on average, farmers would be willing to pay roughly INR 209 for rice seed that has a DT yield profile.^15^ Interestingly, there is some variation in WTP when we look across the different experimental arms. For example, we see that those farmers who were in the DT-only treatment group during kharif 2015 would only be willing to pay about INR 150 for this seed. The greatest WTP for the DT seed is among those in the control group who were not exposed to the DT variety. These farmers would be willing to pay almost INR 80 more than those in the DT treatment group. Similarly, those in the DT-WII group also value the DT seed less than those in the control group (INR 30) and the pooled sample (INR 5). Clearly, since the mean WTPs among those groups who had been exposed to the DT are considerably lower than those who are hearing about it for the first time, it seems plausible that, while there is some sustained enthusiasm for DT, this enthusiasm seems likely to wane as farmers gain some experience with it. While we are unable to explicitly identify the cause of this phenomenon, we suggest that it could arise due to either the slight yield penalty under normal conditions or its inability to protect yields against the full spectrum of drought stress. Some farmers in each of our three sample districts experienced a drought of some degree or another, so it is possible that their experiences with the DT were disappointing. Overall, however, a positive WTP for DT yield across all of these groups seems reasonable given that the three districts in our study area are located in a drought prone region and only 41 per cent of the sample used irrigation on at least one plot at baseline. In the absence of irrigation (which is itself a technological intervention to address drought risk), farmers in our sample seem to highly value the yield distribution component of the risk management product.

Another interesting result emerges when comparing WTP for DT between the two groups that were exposed to DT during kharif 2015. As previously mentioned, those in the DT-only treatment arm would, on average, only be willing to pay roughly INR 150 for the DT seed. Those in the DT-WII treatment arm, however, would be willing to pay more than INR 200 for the DT seed. Given that assignment to these two treatment arms was random, and furthermore that such random assignment attained a significant degree of sample balance on key demographic and economic variables, there seems little in the way of explaining this result other than their experiences with the complementary WII product. In other words, these results suggest that the bundled product increases the value of the DT component above and beyond what it would be valued as an independent product. This finding is consistent with those reported in Ward et al. (2015), who show that positive interaction terms demonstrate the increasing valuation of DT arising due to its packaging with WII, and vice versa.

Though positive and significant the magnitude of WTP for short duration is rather small, averaging roughly INR 10 in the pooled sample. These estimates are in line with anecdotal evidence from the field where farmers reported problems with the DT variety at harvest time. Due to the short duration of Sahbhagi dhan and the long-duration of Swarna, the former matured prior to the latter. Owing to the water levels in the Swarna rice fields, the Sahbhagi dhan crop could not be harvested in a timely manner and this resulted in reported crop losses. Given Swarna’s long duration and the widespread prevalence of its use, the low WTP for the short duration quality is somewhat understandable. In addition, although the short-duration quality theoretically allows earlier establishment of rabi crops – or even cultivating a short duration horticultural crop between the kharif harvest and rabi sowing – the vast majority of the farmers in our study area leave their plots fallow during the rabi season and thus do not reap this benefit.

We estimate that, on average, farmers in the sample are willing to pay INR 263 for limited coverage insurance. This amount is 129–251 per cent greater than the actuarially fair prices (INR 100 for Balasore, INR 115 for Bhadrak, and INR 75 for Mayurbhanj) for this insurance product. This seems high, given that more than 81 percent of the sample did not have life insurance, 94 per cent did not have vehicle insurance, and 97 per cent did not have either health insurance or crop insurance at baseline. But this highlights just how significant the gap in the insurance market in this region really is. A possible explanation for the high WTP for insurance is a disconnect between farmers’ perceived risk and actual risk (Turvey, Gao, Nie, & Wang, 2013). They find that pessimistic farmers (those with more negatively skewed subjective beliefs about risk) are more likely to purchase insurance for their crops. WTP estimates could be upwardly biased due to the hypothetical nature of the experiment however, so these estimates should be treated as a relative measure of utility as opposed to an absolute measure.

The WTP for the limited coverage insurance product is highest among the DT-WII treatment arm, more than INR 40 higher than that in either the DT treatment arm or the control group. This higher valuation, again, suggests that exposure to DT-WII product – perhaps especially the WII component – increases farmers’ valuation for the risk management that the insurance product provides.

These estimates also give us the mean WTP (or, rather since it is negative, willingness to accept, WTA) for basis risk. While the other attributes that we have discussed so far have been discrete (specifically, binary) variables, in which the WTP captured the movement from, e.g., non-tolerant rice to cultivating the DT rice, this particular attribute is treated as a continuous variable, so we can estimate how much, on average, individuals would have to be compensated (e.g., in the form of lower insurance costs, subsidies, etc.) to incur increased basis risk. Specifically, these results suggest that respondents would require a roughly INR 3.6 discount in the cost of insurance for every additional percentage of basis risk. In other words, if basis risk was roughly 35 percent, as estimated by Smith and Watts (n.d.), farmers would, on average, demand a INR 126 discount on the cost of insurance to compensate for incurring this basis risk. Given the derived WTP for the WII products estimated above, however, estimates suggest that farmers would still, on average, be willing to pay more than actuarially fair prices for these WII products, even after factoring in the discount or subsidy that would be required in light of this basis risk. Farmers from the DT-WII treatment arm appear to be the most sensitive to basis risk, requiring the greatest reduction in the cost of insurance. We suggest that this result may arise because some of the farmers in this group may have experienced crop losses due to drought during Kharif 2015 and yet not received insurance payouts. Unpacking this phenomenon and gaining increased insight into farmers’ decisionmaking processes is an ongoing line of inquiry.

In sum, these estimates noticeably point to the fact that farmers have a positive WTP for the different components of the DT-insurance risk management bundle, which further implies a positive WTP for the bundled product (calculated below). While these estimates are based on hypothetical choice scenarios, and may be subject to hypothetical biases, the findings are largely consistent with other stated choice experiments (e.g., Ward et al., 2015). The estimated WTP is perhaps biased upward due to the hypothetical nature of the choice experiment, but nevertheless provide valuable insights into preference rankings.

We assume that WTP for the complete bundled risk management product is simply an additive function of the component WTPs. Under this assumption, and assuming 35 percent basis risk, we estimate that, on average, farmers in our sample would be willing to pay INR 357 for the DT-WII product. On average, farmers in the DT treatment arm are willing to pay the least (INR 290), while farmers in the control group is willing to pay the most (INR 374) for the bundled DT and limited coverage insurance product. This result may reflect some initial enthusiasm for risk management that may be tempered as farmers gain more experience with the two components and experience some of their shortcomings. Across the board, however, these WTPs for the DT-WII product are higher than what would be the actuarially fair cost of the risk management product (i.e., the actuarially-fair cost of the WII plus the cost of the DT seed), and arguably provide strong evidence that the DT-WII product may be a viable solution for managing drought risks in Odisha. And given that those farmers that were in the DT-WII treatment arm have a significantly higher valuation for the product than those that were only exposed to the DT variety during kharif 2015, there appears to be evidence that farmers perceive and highly value the additional and complementary risk management that the insurance component offers.

## 6 Concluding remarks

This study provides early evidence on the potential viability for a bundled risk management product applicable for rainfed agricultural production systems in which drought risks pose a significant threat to farmer livelihoods. The risk management product in question combines the strengths of two recent innovations for agricultural risk management. One of these innovations, DT rice, provides yield benefits relative to non-tolerant rice varieties and hybrids, but mostly under only moderate to severe drought stress levels. Under normal conditions, or if drought stress is too severe, there may not be any noticeable advantages of the DT varieties. Furthermore, even under stressed conditions in which the DT varieties confer yield advantages vis-à-vis non-tolerant varieties, these benefits remain relative, while there remain non-trivial absolute yield declines that still threaten overall farm livelihoods. The other innovation, WII, provides risk management that is generally more affordable than traditional crop insurance, since it does not rely upon field-level assessments of losses, but instead bases insurance payments upon the realization of easily observable weather outcomes. But while the cost of insurance is generally lower than with traditional crop insurance, and there are fewer problems associated with asymmetric information between the insured and the insurer, there is a non-trivial probability that the conditions at the specific location at which weather outcomes are measured is different from the actual weather conditions on farmers’ fields. This, in turn, results in a risk that farmers could experience crop losses on their field (due to drought or otherwise) and yet not receive any payment from the insurer. This condition, known as basis risk, has often been observed to be one of the major constraints inhibiting the success of WII pilot projects around the world.

The bundled product, which consists of a WII product calibrated and optimized to complement the expected benefit profile of the DT rice variety, would provide a more comprehensive benefit profile and would overcome many of the problems of basis risk and other constraints that would limit the risk management potential of either of these two instruments on their own. In this study, we have used a discrete choice experiment to study farmers’ preferences for this bundled risk management product and its sub-components. The early results reported here support the hypothesis that farmers would value this bundled product, and would be willing to pay more than the actuarially fair cost for the complementary product. The present study represents merely the first step in analyzing demand, uptake, and ultimately the impact of this risk management product on farmers’ production and livelihoods. Given that this choice experiment was conducted as part of the multi-year project’s midline survey, there are many questions that remain to be answered.

The results from the present study suggest that demand for the bundled DT-WII product is partly a function of experience with the product and its sub-components. While there appears to be some enthusiasm for the DT variety, for example, the results of the present study suggest that such enthusiasm may wane over time, perhaps due to either the yield penalty under normal conditions or because the variety performs no better than non-tolerant varieties under extreme drought stress. But while this may be the case, our results suggest that being packaged alongside the calibrated WII product increases the value of the DT product, as farmers perceive the intrinsic complementarities between the two components. Furthermore, those farmers who were randomly allocated to the DT-WII treatment arm had significantly higher valuations for both the complementary WII product and the DT variety relative to those who were randomly allocated to the DT only treatment arm, suggesting that experiences with the DT-WII product leads farmers to perceive that each of the two components are more valuable than if farmers were only exposed to the DT.

Our results also suggest that farmers are quite sensitive to basis risk, and would require roughly a INR 125 reduction in the cost of insurance to cope with a 35 percent risk of incurring crop losses and yet not receiving an insurance payout. Many farmers in our sample have incredibly little experience with insurance, if any, so as they gain more experience with insurance, and particularly as they get more experience with index insurance, they are likely to learn more about basis risk and appreciate its significance in their specific contexts. While farmers in the sample were exposed to an experiential learning exercise prior to completing the discrete choice experiment, our results suggest that those farmers who had been randomly allocated to the DT-WII treatment arm during kharif 2015 were significantly more sensitive to basis risk in the choice experiment than farmers in either the DT only treatment arm or those in the comparison group. Since it is likely that some farmers in this group likely experienced the adverse consequences of basis risk first hand, they are likely painfully aware of how pernicious it could be. Yet despite the impacts of basis risk in muting WTP for the complementary DT-WII product, we find considerable evidence that this product may be a viable product to address drought risk management in rainfed production systems in Odisha, and potentially beyond.

## Conflict of interest

None.

## Footnotes

1 In one of the only known experiments studying the relative performance of Sahbhagi dhan and other varieties under drought conditions, Verulkar et al. (2010) suggest that the yield benefits of Sahbhagi dhan relative to other popular varieties persist even under severe drought conditions. We conservatively assume that such relative benefits may accrue on experimental stations, but may not translate onto farmers’ fields. We therefore maintain the assumption of declining relative yield benefits under severe drought stress conditions.

2 The term ‘basis risk’ is derived from the commodity trading literature, in which it refers to the risk of a various forms of contract nonperformance, resulting in the non-convergence of the asset spot price and the futures price on the expiration date of the contract.

3 A crore is a unit of measurement (typically of money or people) commonly used throughout South Asia, equivalent to 10 million.

4 To the best of our knowledge, there have been no published studies evaluating either the uptake of or impact of such an insurance/improved rice variety package, either in India or elsewhere. There is ongoing work involving a structurally-similar insurance and drought-tolerant maize variety in sub-Saharan Africa supported by the BASIS Feed the Future Innovation Lab for Assets and Market Access in collaboration with the International Maize and Wheat Improvement Center (CIMMYT) and the Drought-Tolerant Maize for Africa (DTMA) project.

5 The MSP is the price at which the government buys rice as part of its public procurement initiative. The MSP is determined by the Cabinet Committee on Economic Affairs, based on recommendations from the Commission for Agricultural Costs and Prices, and is meant to reflect costs of production. We assumed that the 2016 MSP would be equivalent to the 2015 MSP plus 8 percent, where this increment is the average percentage increment observed in the historical MSP data dating back to 1970.

6 Although EVT is not widely used in many social sciences, there are very many applications for EVT and the analysis of extremes. EVT is widely used in finance to study, among other things, risk exposure. EVT is also widely used within the physical sciences, particularly hydrology and meteorology. Hydrologists use EVT to study streamflows (e.g., 100-year flood events) and to development protective measures against these extremes. Meteorologists use EVT to study weather extremes, such as extreme temperature and rainfall events, extreme winds, etc.

7 The terms “return level” and “return period” are intimately related concepts that are central to extreme value theory. The return period is an estimate of the expected interval between occurrences of an event of a given magnitude, and is calculated as simply the inverse of the probability of said event occurring during any given year. For example, the return period for a ‘100-year flood’ would be simply 100 years (with associated annual probability of 0.01). The return level is an estimate of the magnitude of the event that would occur with a given return period or probability. In the case of a ‘100-year flood’, the return level might be some measure of the streamflow that would be expected with probability 0.01.

8 To obtain the set $\left\{x_{i}\right\}$, we used daily rainfall data from weather stations in each of the three districts from 1940 – 2011. We then limited our dataset to consider only rainfall observations during the monsoon period (June 15–October 15, inclusive). Days with rainfall less than 2mm (cumulative over the 24 h period) were classified as dry days, and dry spells were then identified as a series of consecutive dry days. We then identified the maximum dry spell that occurred for each year, thus yielding the set $\left\{x_{i}\right\}$. The assumption that the $x_{i}$ are independent may be a strong assumption in some contexts. For the purposes of this hypothetical preference elicitation exercise, we maintain the assumption of independence, though actual implementation of a program such as this would likely want to relax this assumption and estimate a more general model specification that would allow for local temporal dependence (c.f. Leadbetter, Lindgren, & Rootzen, 1983).

9 Given the geographic and agroecological similarities between Balasore and Bhadrak, we assume the probabilities of moderate and severe droughts are the same in the two districts.

10 With any index insurance product, there is likely to be some residual basis risk. For the bundled product, this is still the case. But there is evidence to suggest that the degree of basis risk is declining in the severity of the event that comprises the index trigger, likely because the spatial correlation in yields increases with hazard severity. Skees (2007, p. 6) notes, “if extreme events impact large numbers of households in the same location then basis risk also should be lower for catastrophic loss events.”

11 At the time this study began in January 2015 Bant and Agarpada were two separate blocks. Soon after the project began, they were merged into one block.

12 Allowing the price coefficient to be distributed normally can be problematic for various reasons (e.g., Revelt & Train, 1999; Ubilava & Foster, 2009). One common approach for circumventing these challenges is to simply assume that the price coefficient is fixed, which is the same as assuming that preferences over prices are homogeneous in the population. While this does introduce some advantages, it also introduces the distinct disadvantage of a lack of realism: If we believe that individuals’ preferences vary over all of the other bundle attributes, why should we not believe that individuals’ preferences over money vary as well? An alternative approach that is often advocated is to assume that the (negative) price coefficient is distributed lognormally. This has some appeal, but the fat tails of the lognormal distribution can result in an increased probability of extreme coefficients.

13 Recent advancements in choice modeling allow for model estimation directly in WTP-space (Scarpa, Thiene, & Train, 2008). This approach however, can be computationally-intensive, and can result in non-convergence when estimating models with a large number of observations.

14 Additionally, in irrigated systems, farmers could take advantage of Sahbhagi dhan’s short duration to plant a long duration rabi crop early, which could enable the farmer to harvest this subsequent crop before terminal heat stress set in. Given the limited irrigation facilities and extensive rabi fallows in Odisha, this is not a likely scenario.

15 The area of land that the quantity of DT seed was intended to cultivate was 10 decimals, but it is certainly possible that the respondents had difficulty calculating the quantity of seed that would be required to plant such a small parcel of land, and perhaps simplified the decisionmaking process and focused on a larger quantity of seed for which they are perhaps more accustomed to purchasing.
